# Supplementary material for: Discovery of New Small Molecule Hits as Hepatitis B Virus Capsid Assembly Modulators: Structure and Pharmacophore-Based Approaches
Source: Viruses. 2021 Apr 27;13(5):770. doi: 10.3390/v13050770 (PMC8146408; doi:10.3390/v13050770)
Supplement: Supplementary file 1 [file viruses-13-00770-s001.zip › viruses-1126474-supplementary.pdf]

Supplementary Information

Discovery of New Small Molecule Hits as Hepatitis B Virus Capsid Assembly Modulators: Structure and Pharmacophore-Based Approaches

Sameera Senaweera [1], Haijuan Du [2,3], Huanchun Zhang [2,3], Karen A. Kirby [2,3], Philip R. Tedbury [2,3], Jiashu Xie [1], Stefan G. Sarafianos [2,3] and Zhengqiang Wang [1,*]

1 Center for Drug Design, College of Pharmacy, University of Minnesota, Minneapolis, MN 55455, USA
2 Laboratory of Biochemical Pharmacology, Department of Pediatrics, Emory University School of Medicine, Atlanta, GA 30322, USA
3 Children's Healthcare of Atlanta, Atlanta, GA 30322, USA
* Correspondence: wangx472@umn.edu; Tel.: +1-612-626-7025

Table of Contents

1. General experimental S2
2. Scheme S1: Synthesis of in-house compounds S2
3. NMR spectra S7
4. Table S1: Docking scores of the selected 100 compound from the virtual screening S13
5. Figure S1: Selected TSA curves S32
6. References S33

General Experimental

All commercial chemicals were used as supplied unless otherwise indicated. Flash chromatography was performed on a Teledyne Combiflash RF-200 with RediSep silica columns (silica) and indicated mobile phase. Moisture sensitive reactions were performed under an inert atmosphere of ultrapure argon with oven-dried glassware. ^{1}H and ^{13}C NMR spectra were recorded on a Varian 600 MHz spectrometer. Mass data were acquired on an Agilent TOF II TOS/MS spectrometer capable of ESI and APCI ion sources.

Scheme 1. Synthesis of in-house compounds.

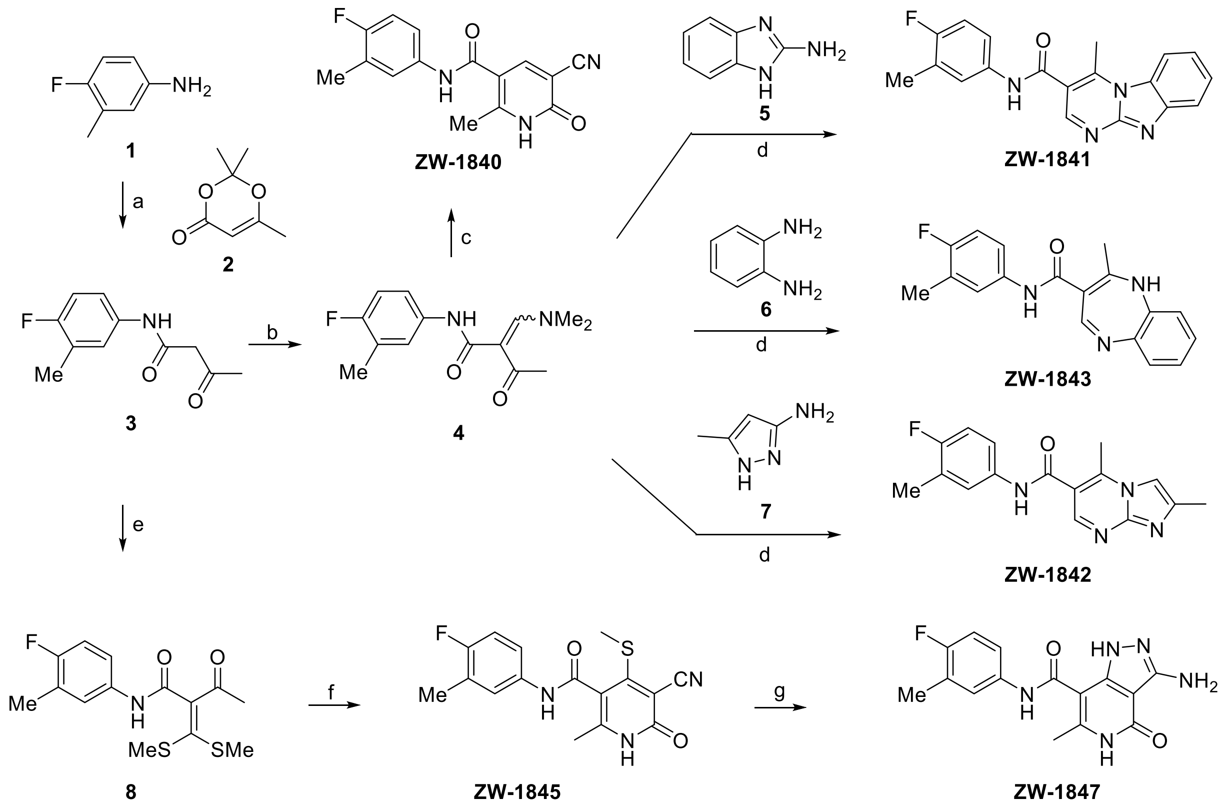

Reagents and conditions: a) Water, reflux; b) DMF-DMA, xylenes; c) Ethyl cyanoacetate, NaOEt, EtOH; d) RNH_2, pyridine, reflux; e) NaO^tBu, CS_2, THF then, MeI ; f) Cyanoacetate, NaO^iPr, ^iPrOH, reflux; g) NH_2NH_2, ^iPrOH, reflux

Synthesis of intermediate **9** (*N*-(4-fluoro-3-methylphenyl)-3-oxobutanamide)

Compound **9** was synthesized using a modified literature procedure [1]. In a round bottom flask, 4-fluoro-3-methylaniniline (5.0 g, 40 mmol) and DI water (120 mL) was heated to reflux. Upon reflux, TMD (2,2,6-Trimethyl-4*H*-1,3-dioxin-4-one) (1.5 equiv) was added and the heating was maintained for another 2.5 h. Upon completion of the reaction as determined by TLC (Hex:EtOAc 1:1), the reaction mixture was allowed to cool and it was extracted with EtOAc (2x). Combined organic phase was dried with Na_2SO_4, filtered and concentrated. The crude was purified by silica gel column chromatography eluting with hexane and EtOAc to obtain compound **9** as an orange oil (yield = 68%). ^{1}H NMR (600 MHz, Chloroform-*d*) δ 9.04 (s, 1H), 7.38 (dd, *J* = 6.2, 3.1 Hz, 1H), 7.29 (dd, *J* = 7.8, 3.4 Hz, 1H), 6.94 (t, *J* = 10.1 Hz, 1H), 3.58 (s, 2H), 2.33 (s, 3H), 2.26 (s, 3H).

Synthesis of intermediate **10** (2-((dimethylamino)methylene)-*N*-(4-fluoro-3-methylphenyl)-3-oxobutanamide)

In a round bottom flask, intermediate **9** (2.5 g, 1.0 equiv) and DMF-DMA (1.0 equiv) was heated to 145 ^{o}C for 3 h. Then, the reaction was cooled down to room temperature. Overtime a turbidity appeared. The solvent was removed, and the resulted orange yellow solid was washed with ether (2x) and it was dried to obtain compound **10** as a white solid (yield = 74%). ^{1}H NMR (600 MHz, Chloroform-*d*) δ 10.51 (s, 1H), 7.68 (s, 1H), 7.44 (d, *J* = 7.0 Hz, 1H), 7.37 (dd, *J* = 8.1, 3.6 Hz, 1H), 6.92 (t, *J* = 9.1 Hz, 1H), 3.19 (br. s, 6H), 2.29 (s, 3H), 2.25 (s, 3H).

Synthesis of ZW-1840 (5-cyano-*N*-(4-fluoro-3-methylphenyl)-2-methyl-6-oxo-1,6-dihydropyridine-3-carboxamide)

ZW-1840 was synthesized using a modified literature procedure [2]. In a two-necked round bottom flask equipped with a reflux condenser, NaOEt (1.0 equiv) was added EtOH (1.0 mL) followed by cyanoacetamide (1.0 equiv) under Ar at room temperature. Then, a solution of compound **10** (100 mg, 1.0 equiv) in EtOH (1.5 mL) was added dropwise. The reaction was allowed to stir for 15 min before it was refluxed for 1.5 h. After the reaction time, ice water was added to the reaction and it was acidified with 2 M HCl. The resulting precipitate was collected by filtration. The solid crude was purified by silica gel column chromatography eluting with DCM and MeOH to obtain ZW-1840 as an off-white solid (yield = 35%). ^{1}H NMR (600 MHz, DMSO-d6) δ 10.14 (br. s, 1H), 8.60 (s, 1H), 8.02 (br. s, 1H), 7.35 (t, J = 9.0 Hz, 1H), 7.28 (dd, J = 6.8, 2.6 Hz, 1H), 7.19 (dt, J = 7.7, 3.6 Hz, 1H), 2.40 (s, 3H), 2.29 (s, 3H). ^{13}C NMR (101 MHz, DMSO-d_6) δ 193.99, 169.52, 162.17, 160.86, 159.74, 157.62, 143.58, 132.06 (d, *J* = 5.9 Hz), 130.70 (d, *J* = 3.3 Hz), 128.34 (d, *J* = 9.0 Hz), 127.18 – 116.32 (m), 110.68, 92.77, 30.57, 14.21 (d, *J* = 3.1 Hz). HRMS (ESI$^-$) *m/z* calcd for $C_{15}H_{11}FN_3O_2$ 284.0841, found 284.0830.

Synthesis of intermediate **14** (2-(bis(methylthio)methylene)-*N*-(4-fluoro-3-methylphenyl)-3-oxobutanamide)

Intermediate **14** was synthesized following a literature procedure [3]. In a two-necked round bottom flask, to a suspension of NaO^{t}Bu (2.0 equiv) in THF (1.5 mL) at 0 ^{o}C was added a solution of CS2 (1.0 equiv) and intermediate **9** (0.5 g, 1.0 equiv) in THF (3.0 mL) over 15 min. After completion of the addition, the mixture was stirred at 0 ^{o}C for another 1 h. Overtime, the

solution turned red. To this, a solution of MeI (2.0 equiv) in THF (1.0 mL) was added dropwise at 0 ^{o}C. The resulting mixture was allowed to warm up to room temperature and it was stirred for another 5 h at room temperature. The mixture was then poured onto crushed ice under stirring. The separated solid was collected by filtration, washed with water (2x), dried in vacuo and crystallized from chloroform to furnish the intermediate **14** as a yellow solid (yield = 70%). ^{1}H NMR (600 MHz, Chloroform-*d*) δ 8.18 (s, 1H), 7.47 (d, *J* = 6.3 Hz, 1H), 7.31 (dd, *J* = 8.2, 4.1 Hz, 1H), 6.96 (t, *J* = 8.9 Hz, 1H), 2.49 (s, 3H), 2.47 (br. s, 6H), 2.27 (s, 3H).

Synthesis of compound ZW-1845 (5-cyano-*N*-(4-fluoro-3-methylphenyl)-2-methyl-4-(methylthio)-6-oxo-1,6-dihydropyridine-3-carboxamide)

ZW-1845 was synthesized following a modified literature procedure [4]. In a two-necked round bottom flask, under Ar, Na^{i}OPr was prepared by dissolving Na (1.0 equiv) in ^{i}PrOH (5.0 mL). The mixture was added cyanoacetamide (1.0 equiv) followed by dropwise addition of compound **14** (0.5 g, 1.0 equiv) in ^{i}PrOH over 15 min. The resulting mixture was further stirred at room temperature for 15 min and heated to reflux for overnight. Upon completion of the reaction as determined by TLC (Hex:EtOAc 7:3), the solvent was evaporated and the resulting solid was treated with 1 N HCl solution to obtain a solid. The solid was filtered, washed with water, and dried to afford ZW-1845 as a yellow solid (yield = 60%). ^{1}H NMR (600 MHz, DMSO-d_6) δ 10.63 (s, 1H), 8.93 (s, 1H), 8.38 (d, *J* = 8.5 Hz, 1H), 7.93 (d, *J* = 8.2 Hz, 1H), 7.68 (dd, *J* = 7.2, 2.6 Hz, 1H), 7.64 (t, *J* = 7.7 Hz, 1H), 7.56 (dt, *J* = 8.0, 3.4 Hz, 1H), 7.47 (t, *J* = 7.8 Hz, 1H), 7.17 (t, *J* = 9.2 Hz, 1H), 3.24 (s, 3H), 2.26 (d, *J* = 2.0 Hz, 3H). ^{13}C NMR (101 MHz, DMSO) δ 163.3, 155.9, 154.3, 150.5, 150.1, 144.7, 134.9 (d, J = 2.9 Hz), 128.3, 126.3, 124.4 (d, J = 18.1 Hz), 123.0 (d, J = 4.8 Hz), 122.0, 119.5, 119.2 (d, J = 7.7 Hz), 116.9, 116.8, 115.1 (d, J = 23.5 Hz), 18.2, 14.4 (d, J = 3.1 Hz). HRMS (ESI^{-}) *m/z* calcd for $C_{16}H_{13}FN_3O_2S$ 330.0718, found 330.0726.

Synthesis of compound ZW-1847 (3-amino-*N*-(4-fluoro-3-methylphenyl)-6-methyl-4-oxo-4,5-dihydro-1*H*-pyrazolo[4,3-*c*]pyridine-7-carboxamide) [4]

In a 0.5-2.0 mL microwave vial equipped with a magnetic stirrer, ZW-1845 (50 mg, 1.0 equiv) was dissolved in ^{i}PrOH (0.6 mL). Then, hydrazine monohydrate (20.0 equiv) was added. The vial was sealed, and the reaction was carried out in a microwave reactor (Biotage Initiator^{+}) at 120 ^{o}C for 40 min. Upon cooling down to room temperature, a solid product appeared in the reaction. The solid was separated and washed with MeOH (2x) followed by ether (2x) and dried

to furnish ZW-1847 as an off-white solid (yield = 60%). ^{1}H NMR (600 MHz, DMSO-d_6) δ 10.64 (s, 2H), 7.56 (d, *J* = 7.0 Hz, 1H), 7.46 (dt, *J* = 7.6, 3.5 Hz, 1H), 7.10 (t, *J* = 9.1 Hz, 1H), 6.03 (s, 2H), 2.46 (s, 3H), 2.23 (s, 3H). ^{13}C NMR (101 MHz, DMSO-d_6) δ 163.7, 160.5, 155.9, 153.7, 149.3 (d, *J* = 43.9 Hz), 142.7, 135.7, 124.7, 122.9, 119.3 (d, *J* = 51.2 Hz), 115.4 (d, *J* = 22.4 Hz), 101.2, 95.1, 19.1, 14.8 (d, *J* = 3.1 Hz). HRMS (ESI$^-$) *m/z* calcd for $C_{15}H_{13}FN_5O_2$ 314.1059, found 314.1045.

General method A for the synthesis of compounds 3-5

Compounds 3-5 were prepared following a literature procedure [5]. In a pressure vial equipped with a stir bar, intermediate **10** (100 mg, 1.0 equiv) was added pyridine (1.0 mL) followed by the corresponding amine (1.0 equiv). The vial was sealed, and it was heated at 110 °C for overnight. Overtime, a solid appeared. After the reaction time it was cooled to room temperature. The solid was allowed to settle down and the liquid layer (pyridine) was separated. The solid was washed with ether (3x) followed by hot ether (1x) and dried to afford the final compounds as white solids (yield = 45 - 65%).

Synthesis of ZW-1841 (*N*-(4-fluoro-3-methylphenyl)-4-methylbenzo[4,5]imidazo[1,2-*a*]pyrimidine-3-carboxamide)

ZW-1841 was synthesized following the general method A using 2-aminobenzimidazole as the amine. ^{1}H NMR (600 MHz, DMSO-d_6) δ 10.63 (s, 1H), 8.93 (s, 1H), 8.38 (d, *J* = 8.5 Hz, 1H), 7.93 (d, *J* = 8.2 Hz, 1H), 7.68 (dd, *J* = 7.2, 2.6 Hz, 1H), 7.64 (t, *J* = 7.7 Hz, 1H), 7.56 (dt, *J* = 8.0, 3.4 Hz, 1H), 7.47 (t, *J* = 7.8 Hz, 1H), 7.17 (t, *J* = 9.2 Hz, 1H), 3.24 (s, 3H), 2.26 (d, *J* = 2.0 Hz, 3H). ^{13}C NMR (101 MHz, DMSO-d_6) δ 163.3, 155.9, 154.3, 150.5, 150.1, 144.7, 134.9 (d, *J* = 2.9 Hz), 128.3, 126.3, 124.4 (d, *J* = 18.1 Hz), 123.0 (d, *J* = 4.8 Hz), 122.0, 119.5, 119.2 (d, *J* = 7.7 Hz), 116.9, 116.8, 115.1 (d, *J* = 23.5 Hz), 18.2, 14.4 (d, *J* = 3.1 Hz). HRMS (ESI$^-$) *m/z* calcd for $C_{19}H_{14}FN_4O$ 333.1157, found 333.0049.

Synthesis of ZW-1843 (*N*-(4-fluoro-3-methylphenyl)-2-methyl-1*H*-benzo[*b*][1,4]diazepine-3-carboxamide)

ZW-1843 was synthesized following the general method A using *o*-Phenylenediamine as the amine.

^{1}H NMR (600 MHz, DMSO-d_6) δ 12.27 (d, *J* = 12.8 Hz, 1H), 11.94 (s, 1H), 8.49 (d, *J* = 12.8 Hz, 1H), 7.52 (dd, *J* = 7.1, 2.7 Hz, 1H), 7.48 (ddd, *J* = 7.8, 4.5, 2.7 Hz, 1H), 7.40 (dd, *J* = 8.1, 1.4 Hz, 1H), 7.09 (t, *J* = 9.1 Hz, 1H), 7.00 (td, *J* = 7.6, 1.4 Hz, 1H), 6.87 (dd, *J* = 8.0, 1.4 Hz, 1H), 6.74 (td, *J* = 7.6, 1.4 Hz, 1H), 5.02 (s, 2H), 2.44 (s, 3H), 2.23 (d, *J* = 1.9 Hz, 3H). ^{13}C NMR (101 MHz, DMSO) δ 197.1, 167.3, 158.3, 156.5, 156.0, 140.5, 134.9 (d, *J* = 2.8 Hz), 127.4, 126.9, 123.3 (d, *J* = 4.6 Hz), 125.4 – 114.6 (m), 119.6, 119.5 (d, *J* = 7.7 Hz), 118.6, 117.6, 103.1, 27.0, 14.7 (d, *J* = 3.1 Hz). HRMS (ESI$^-$) *m/z* calcd for $C_{18}H_{17}FN_3O_2$ 326.1310, found 326.1298.

<u>Synthesis of ZW-1842 (*N*-(4-fluoro-3-methylphenyl)-2,5-dimethylimidazo[1,2-*a*]pyrimidine-6-carboxamide)</u>

ZW-1842 was synthesized following the general method A using 3-amino-5-methylpyrazole as the amine. ^{1}H NMR (600 MHz, DMSO-d_6) δ 10.52 (s, 1H), 8.62 (s, 1H), 7.64 (d, *J* = 6.7 Hz, 1H), 7.55 – 7.51 (m, 1H), 7.15 (t, *J* = 9.1 Hz, 1H), 6.64 (s, 1H), 2.86 (d, *J* = 1.4 Hz, 3H), 2.49 (s, 3H), 2.25 (s, 3H). ^{13}C NMR (101 MHz, DMSO-d_6) δ 163.3, 157.1 (d, *J* = 239.8 Hz), 155.6, 148.4, 147.7, 145.8, 134.9 (d, *J* = 2.8 Hz), 124.4 (d, *J* = 18.1 Hz), 123.0 (d, *J* = 4.5 Hz), 119.3 (d, *J* = 8.0 Hz), 116.4, 115.1 (d, *J* = 23.1 Hz), 96.5, 14.7, 14.6, 14.4 (d, *J* = 3.1 Hz). HRMS (ESI$^-$) *m/z* calcd for $C_{16}H_{14}FN_4O$ 297.1157, found 297.1152.

ZW-1840 (5-cyano-*N*-(4-fluoro-3-methylphenyl)-2-methyl-6-oxo-1,6-dihydropyridine-3-carboxamide)

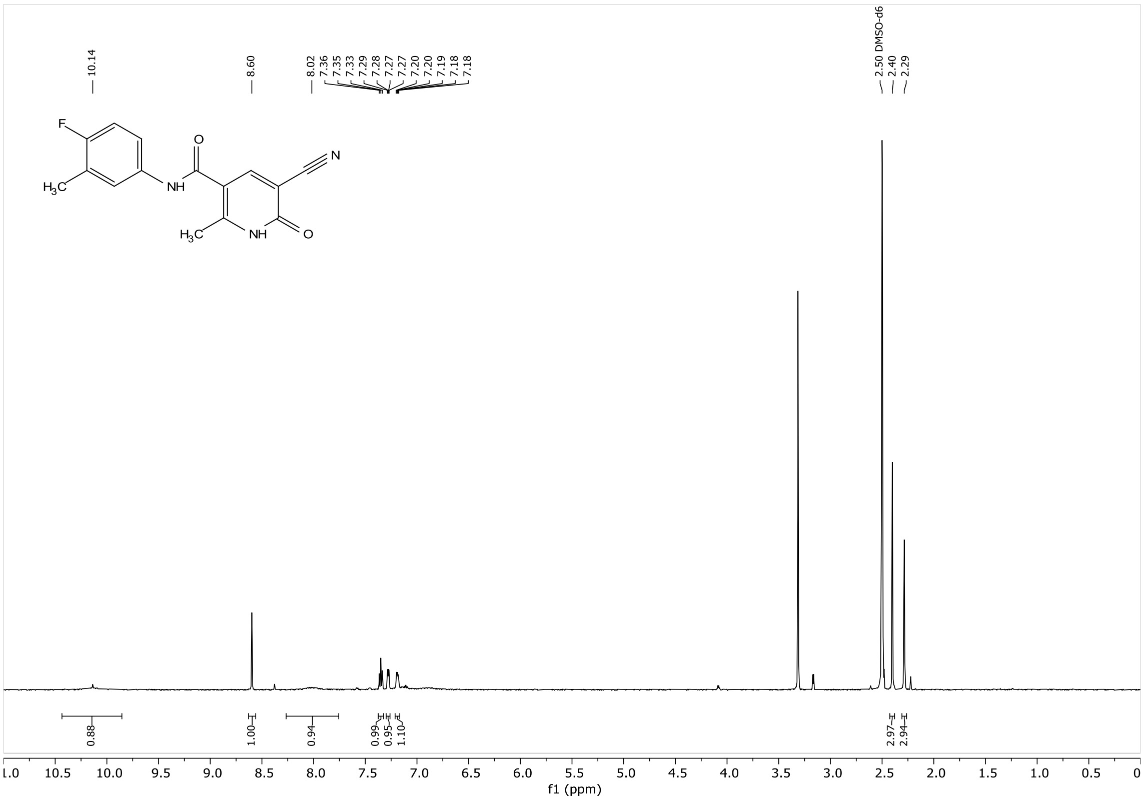
10.14
8.60
8.02
7.36
7.35
7.33
7.29
7.28
7.27
7.27
7.20
7.20
7.19
7.18
7.18
2.50 DMSO-d6
2.40
2.29
F
O
N
H3C
NH
H3C
NH
O
0.88
1.00
0.94
0.99
0.95
1.10
2.97
2.94
f1 (ppm)

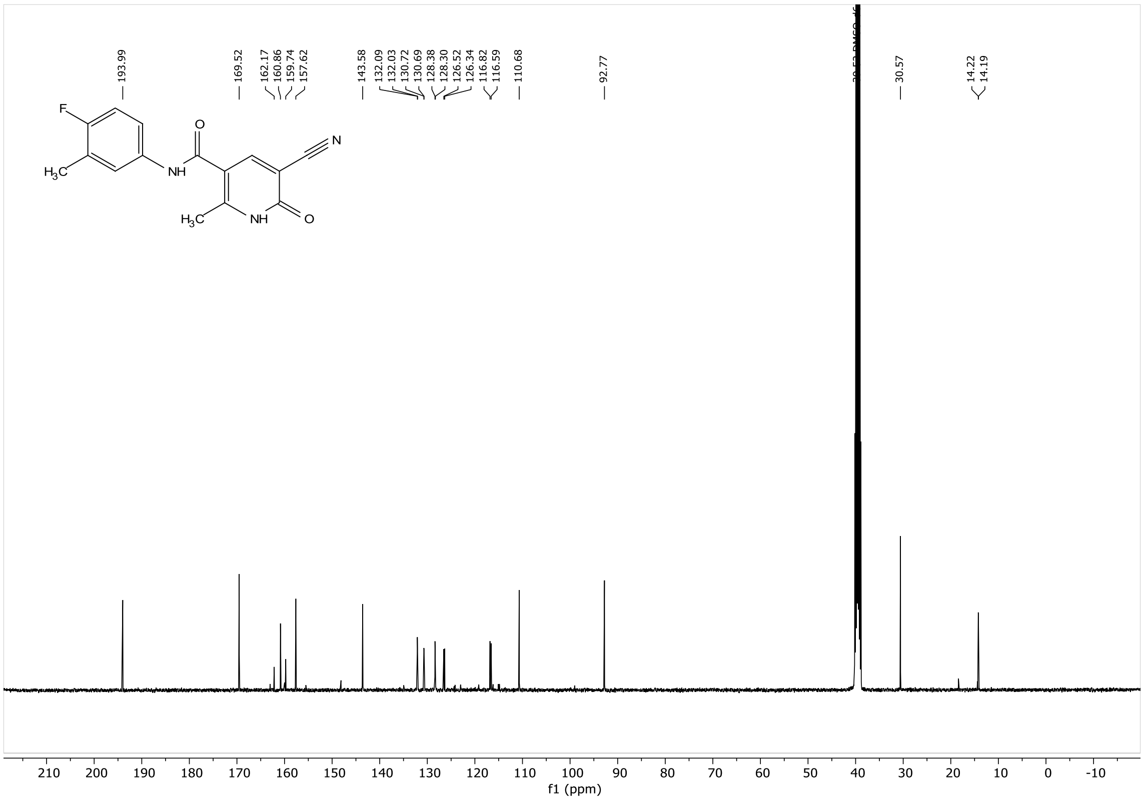
193.99
169.52
162.17
160.86
159.74
157.62
143.58
132.09
132.03
130.72
130.69
128.38
128.30
126.52
126.34
116.82
116.59
110.68
92.77
30.57
14.22
14.19
F
O
N
H3C
NH
H3C
NH
O
f1 (ppm)

ZW-1841 (*N*-(4-fluoro-3-methylphenyl)-4-methylbenzo[4,5]imidazo[1,2-*a*]pyrimidine-3-carboxamide)

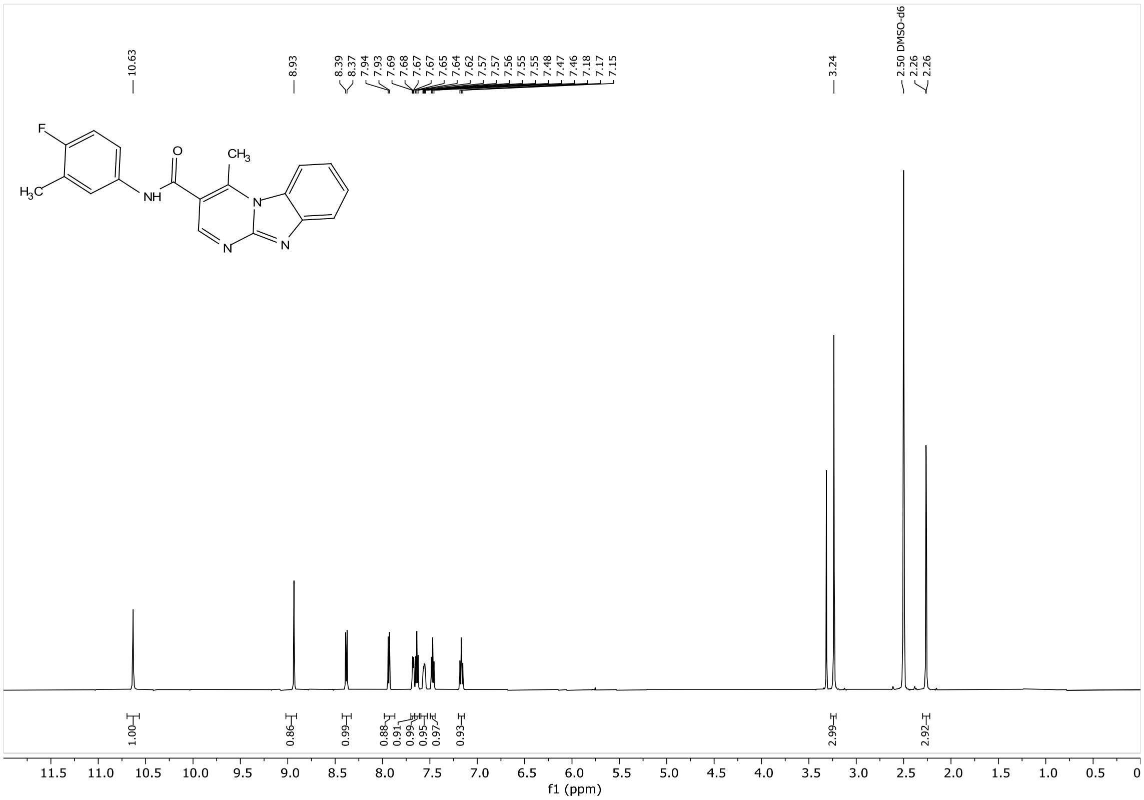

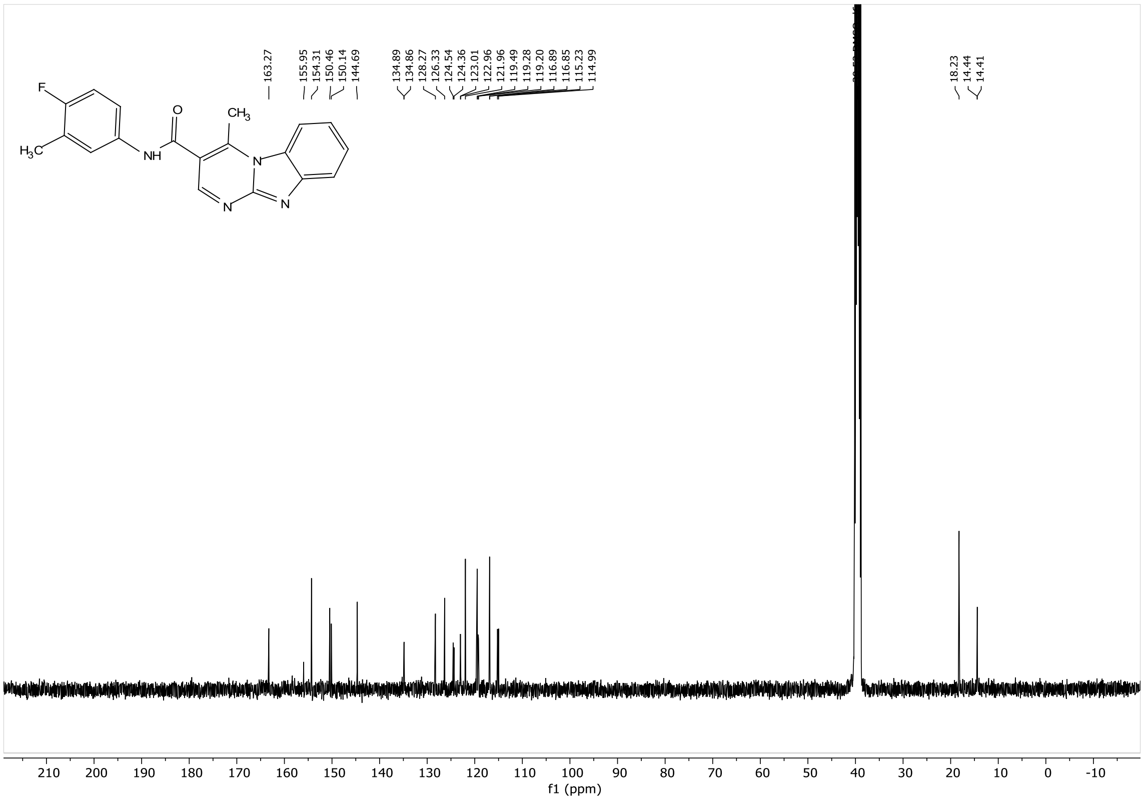

ZW-1842 (*N*-(4-fluoro-3-methylphenyl)-2,5-dimethylimidazo[1,2-*a*]pyrimidine-6-carboxamide)

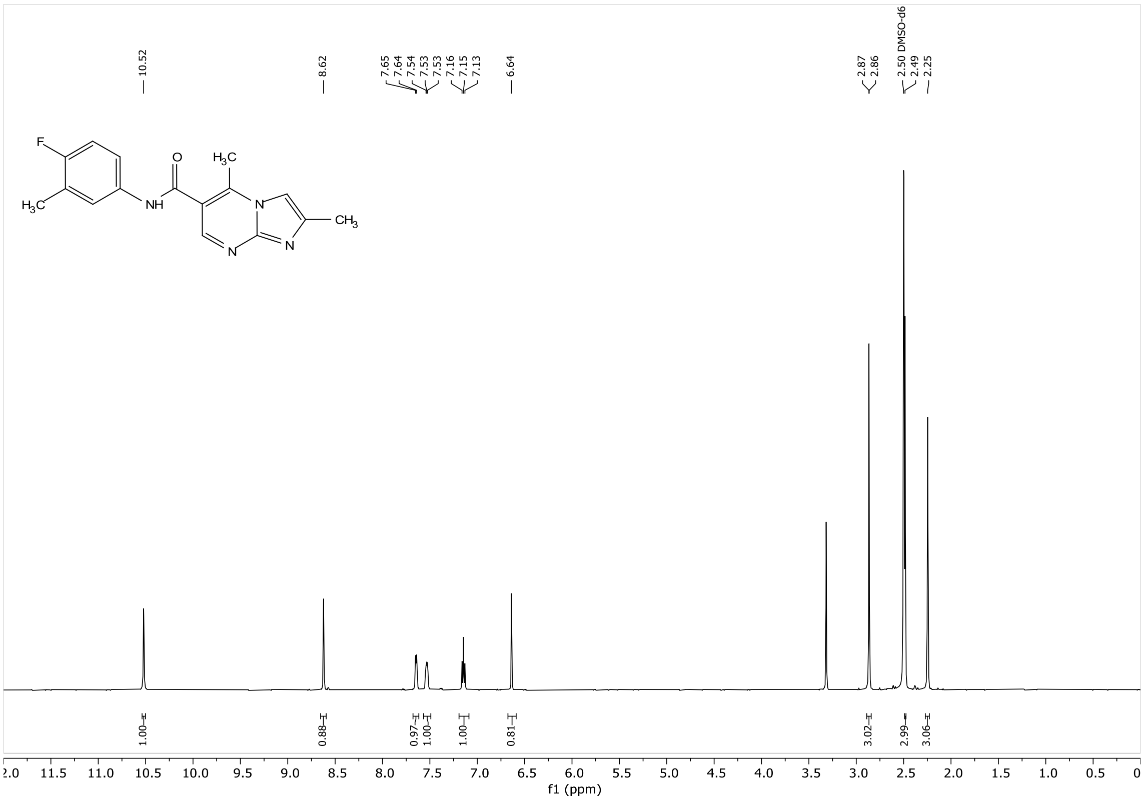

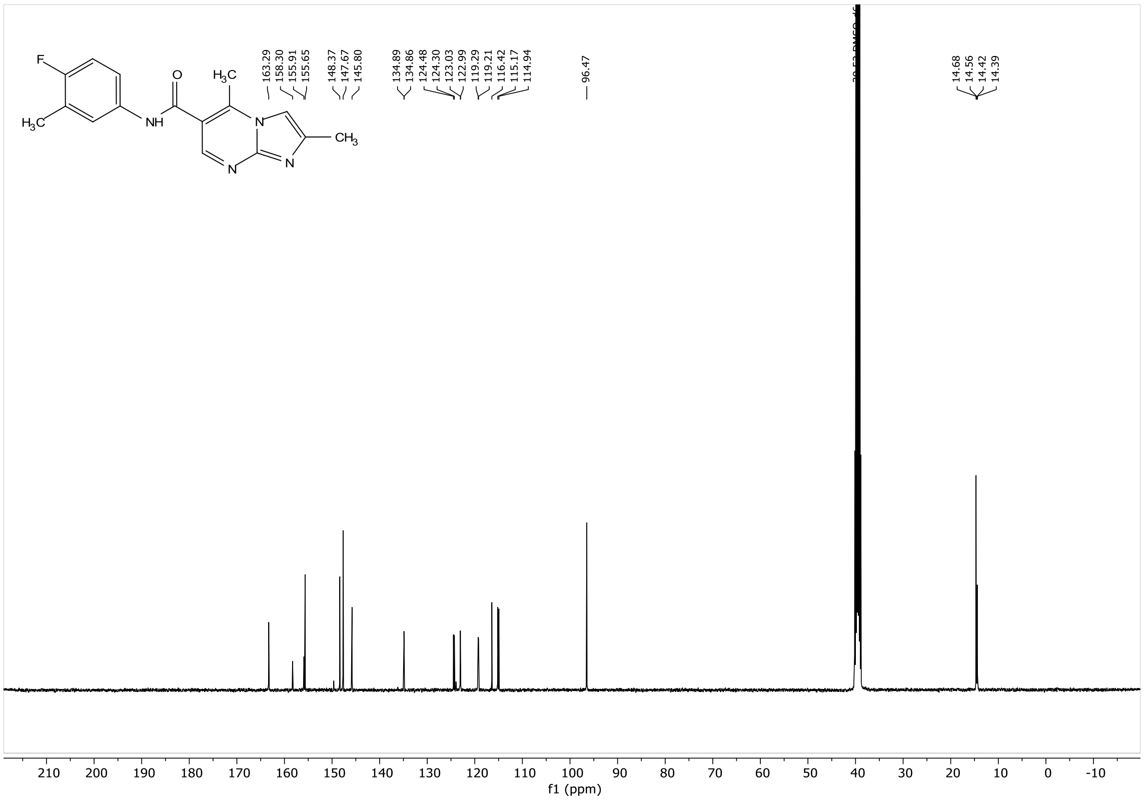

ZW-1843 (*N*-(4-fluoro-3-methylphenyl)-2-methyl-1*H*-benzo[*b*][1,4]diazepine-3-carboxamide)

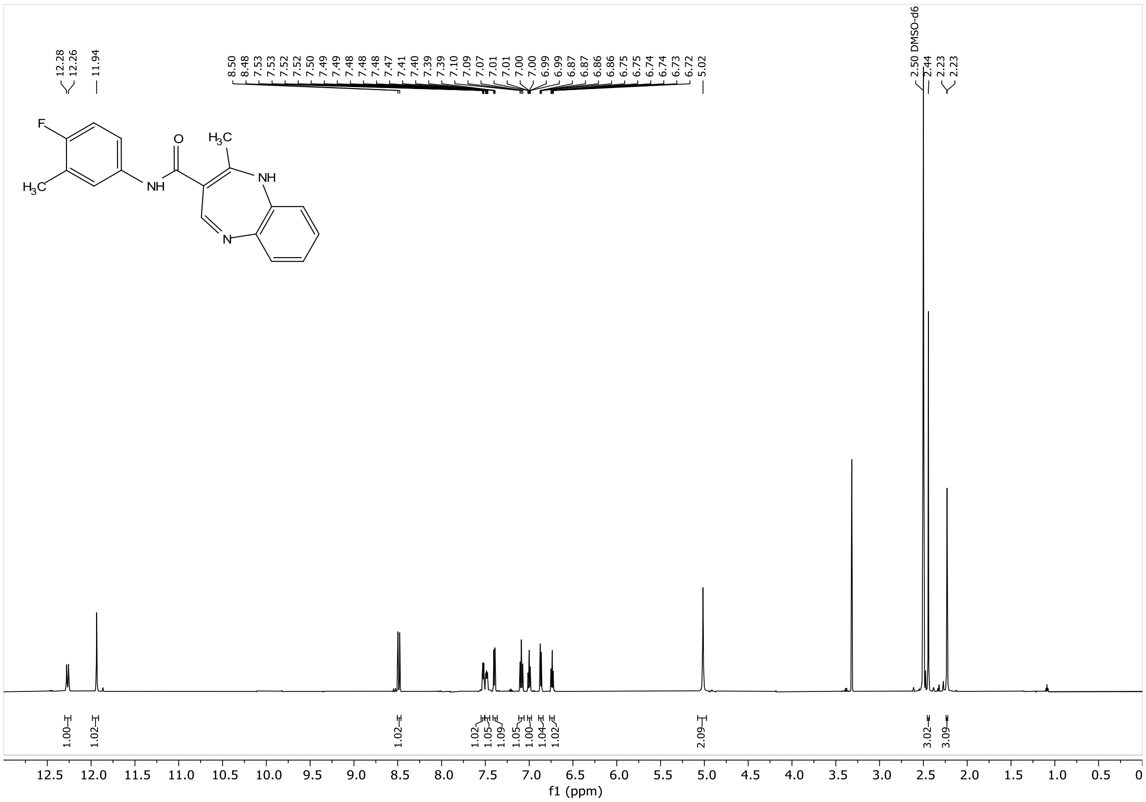

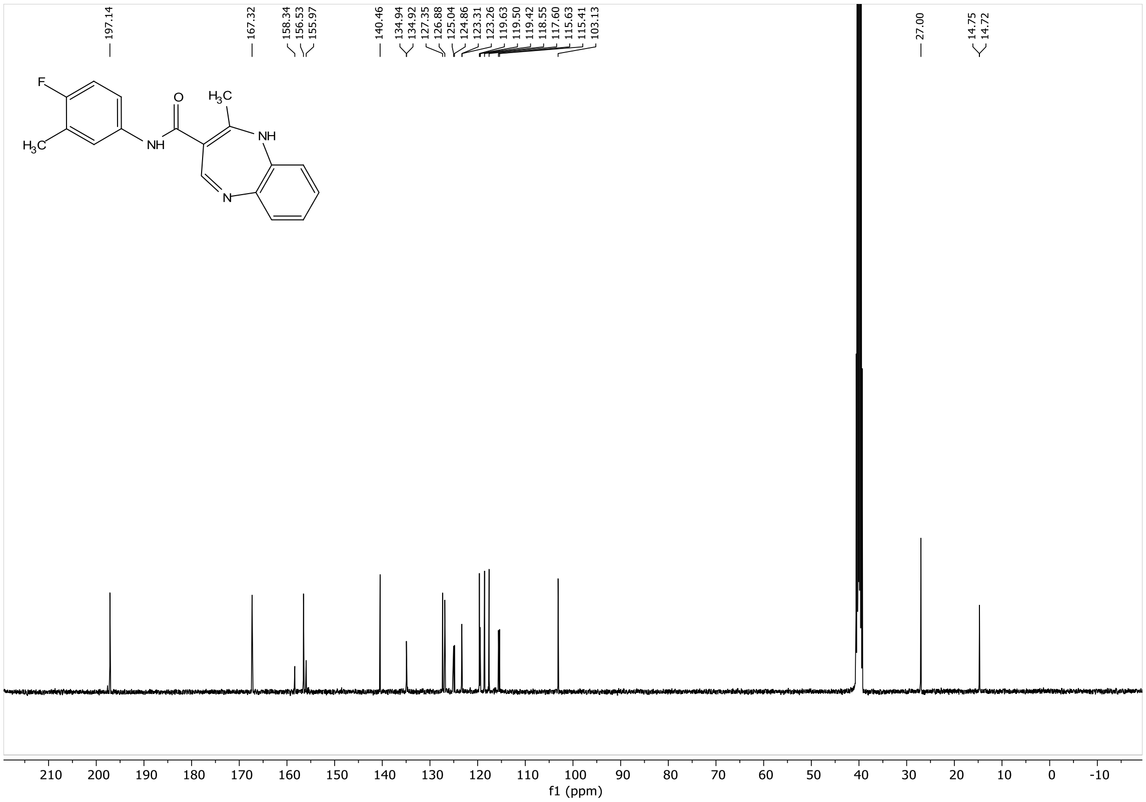

ZW-1845 (5-cyano-*N*-(4-fluoro-3-methylphenyl)-2-methyl-4-(methylthio)-6-oxo-1,6-dihydropyridine-3-carboxamide)

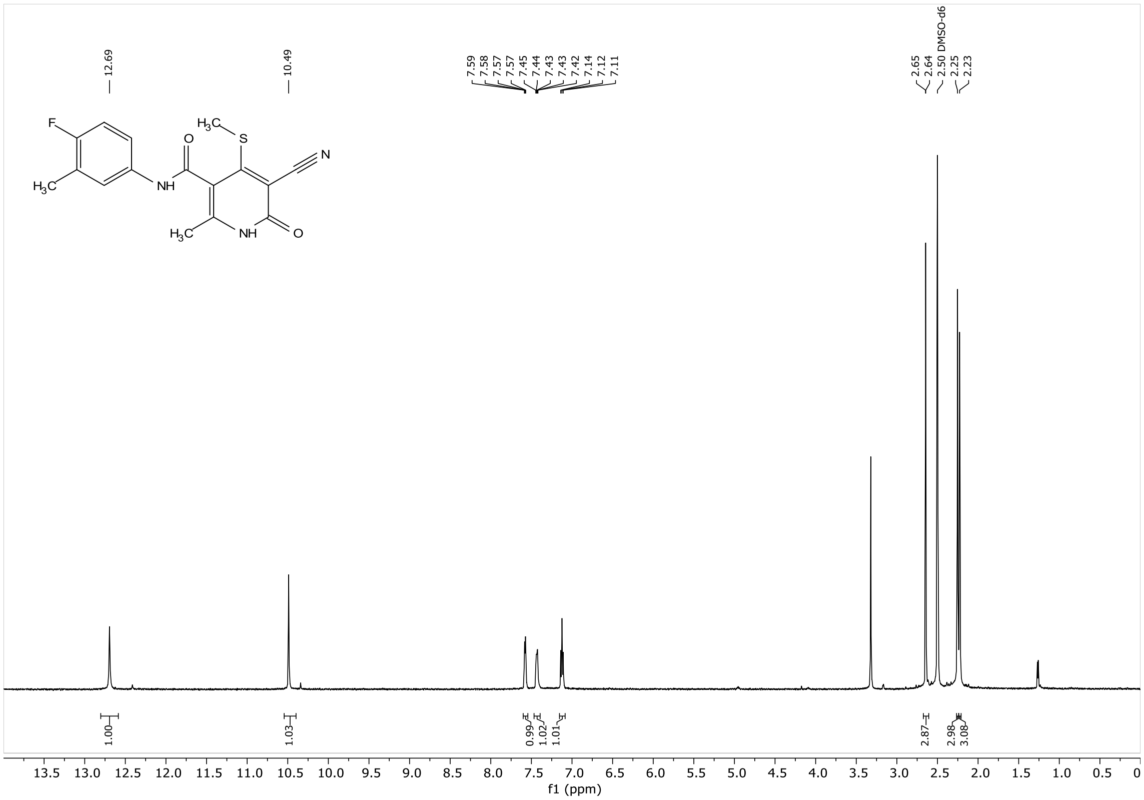

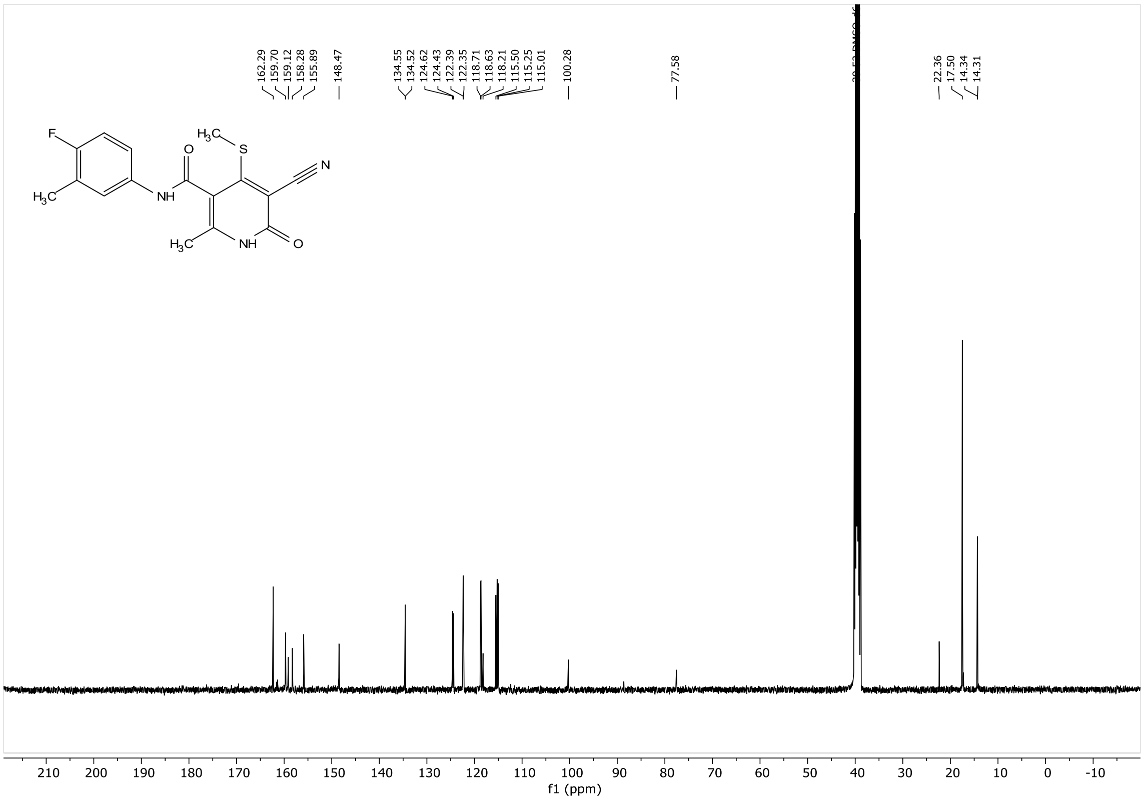

ZW-1847 (3-amino-*N*-(4-fluoro-3-methylphenyl)-6-methyl-4-oxo-4,5-dihydro-1*H*-pyrazolo[4,3-*c*]pyridine-7-carboxamide)

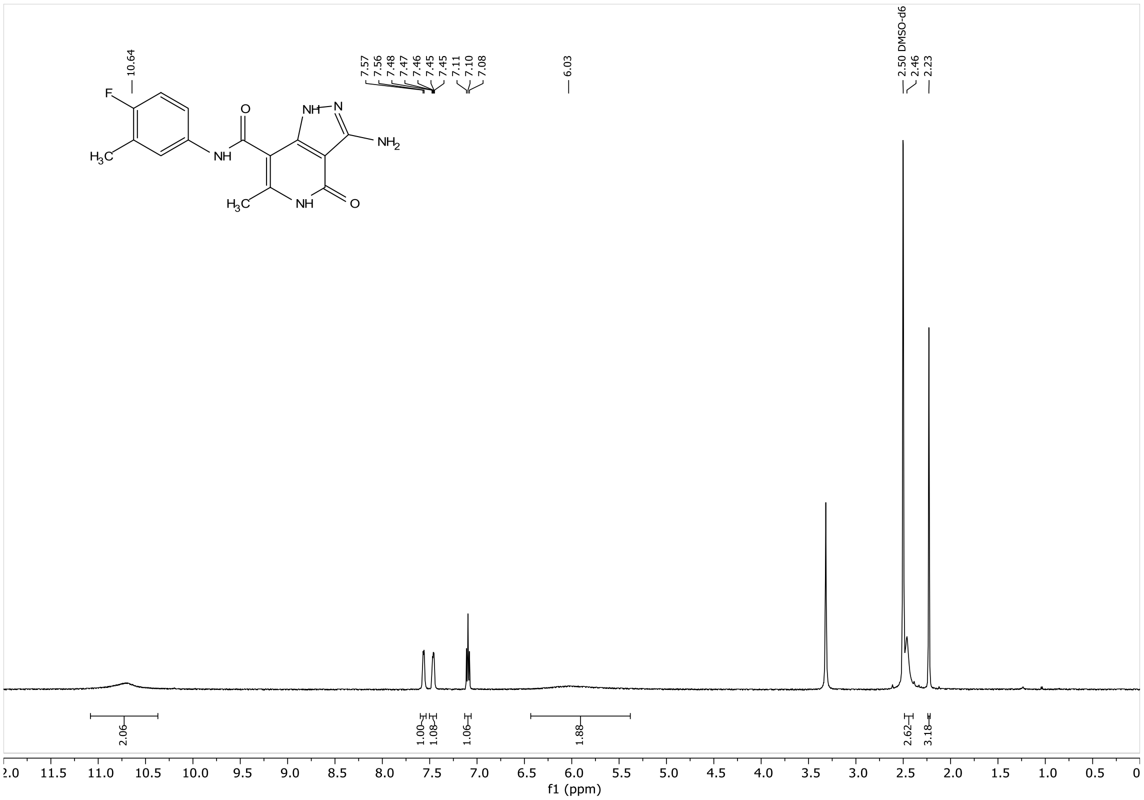

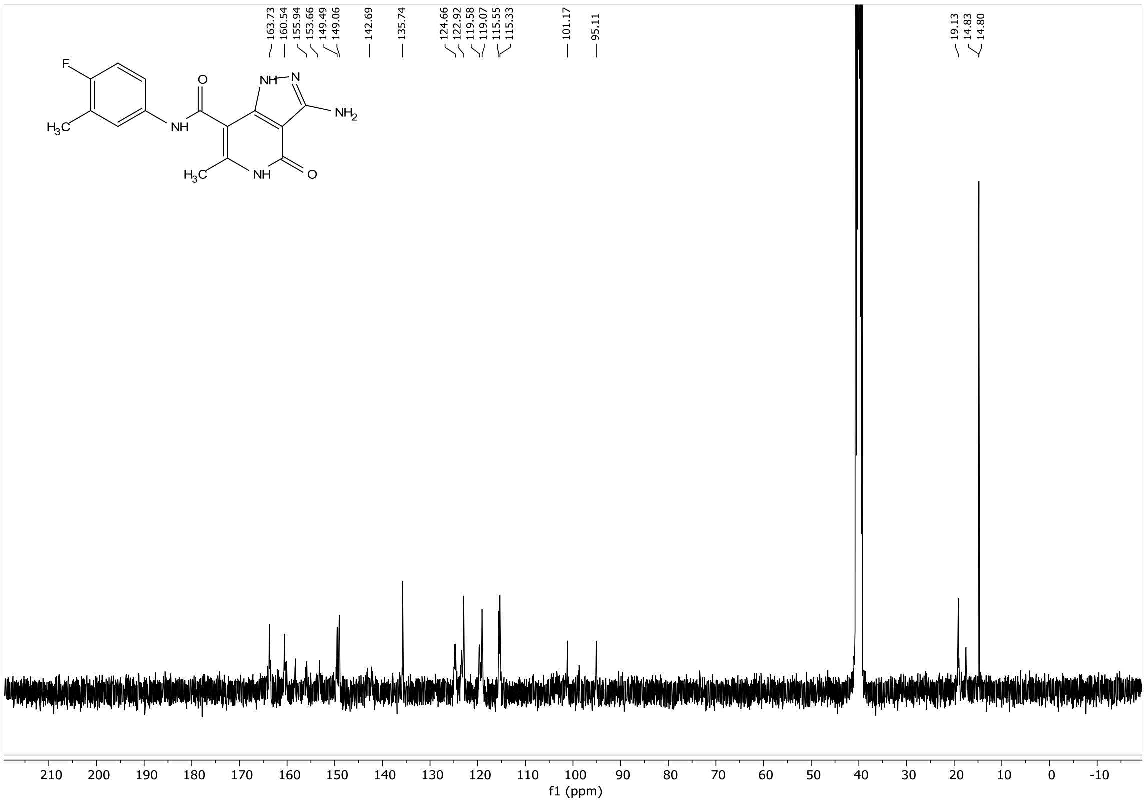

Table S1: Docking scores of the selected 100 compound from the virtual screening

Compound ID	Enamine ID	Structure	Docking score
ZW-1872	Z30720891	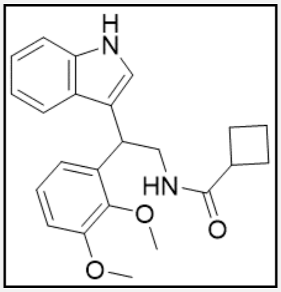	-9.326
ZW-1873	Z82223107	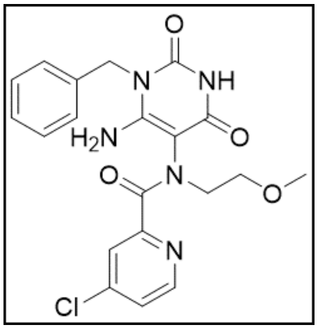	-8.153
ZW-1874	Z16336550	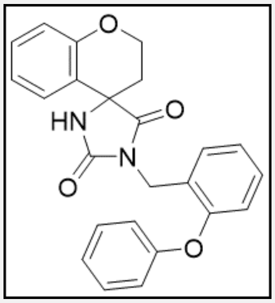	-9.199
ZW-1875	Z94795453	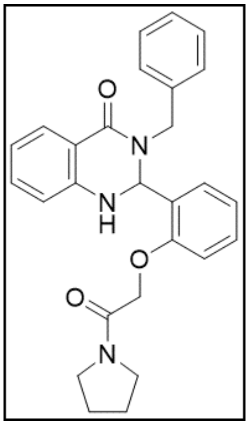	-9.525

ZW-1876	Z109819830	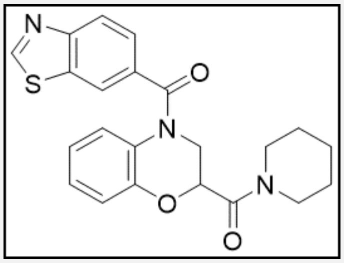	-9.844
ZW-1877	Z127592722	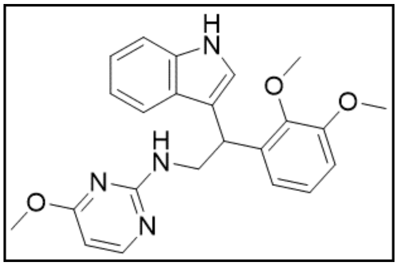	-9.57
ZW-1878	Z191910196	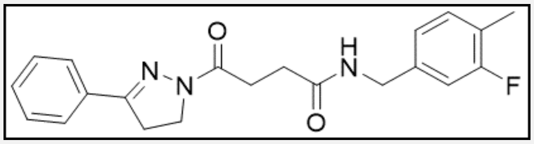	-8.239
ZW-1879	Z94796944	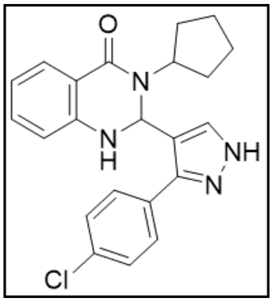	-8.628
ZW-1880	Z26394009	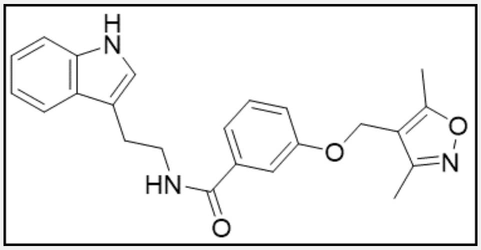	-8.566

ZW-1881	Z229022754	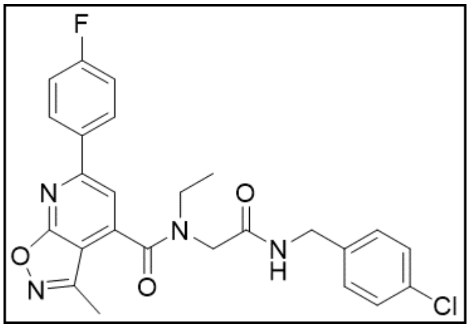	-8.638
ZW-1882	Z28486756	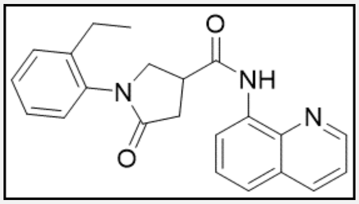	-9.02
ZW-1883	Z107734680	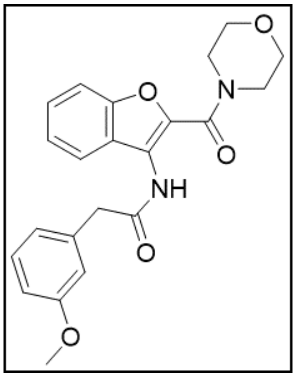	-9.893
ZW-1884	Z641840076	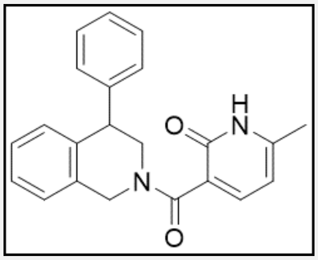	-9.874
ZW-1885	Z454209352	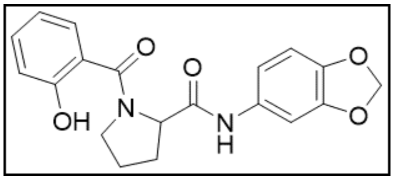	-9.413

ZW-1886	Z823355794	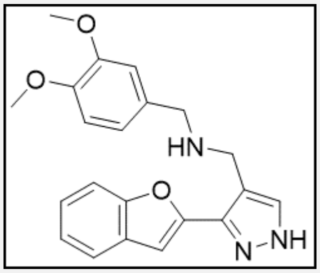	-8.121
ZW-1887	Z290050640	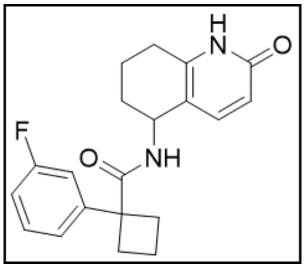	-8.342
ZW-1888	Z1128771581	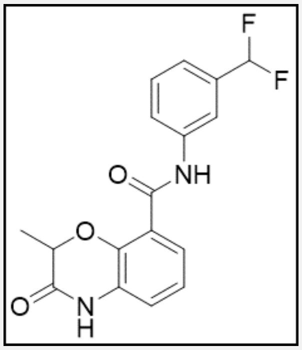	-9.168
ZW-1889	Z25701711	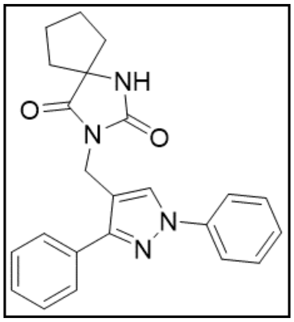	-9.815
ZW-1890	Z106551590	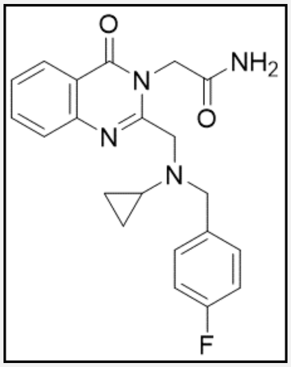	-9.481

ZW-1891	Z107737122	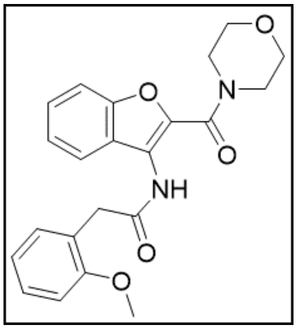	-8.749
ZW-1892	Z222959730	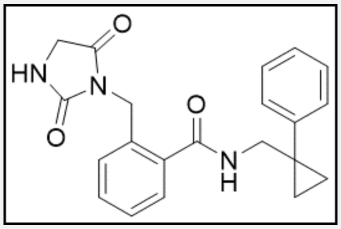	-8.363
ZW-1893	Z423526932	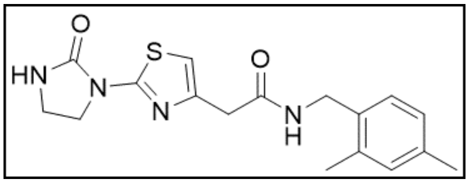	-8.532
ZW-1894	Z70997275	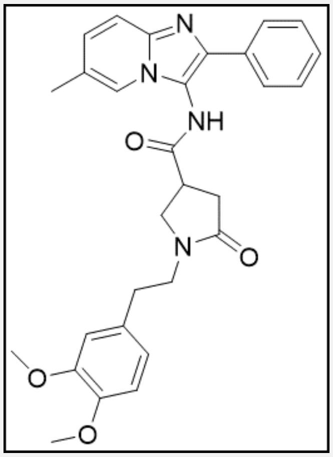	-9.486
ZW-1895	Z737544166	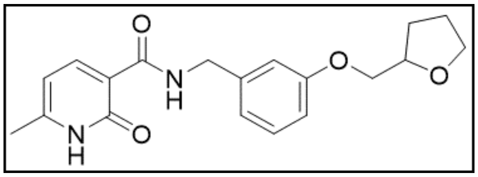	-9.634

ZW-1896	Z823391464	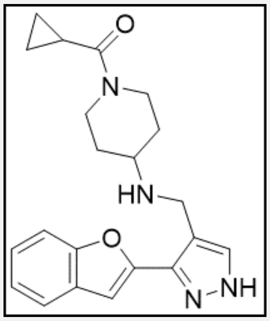	-8.761
ZW-1897	Z775154976	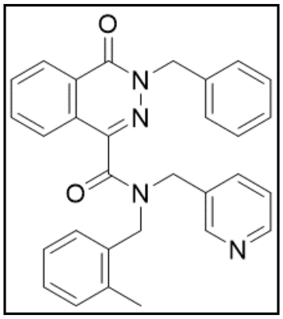	-9.348
ZW-1898	Z31837053	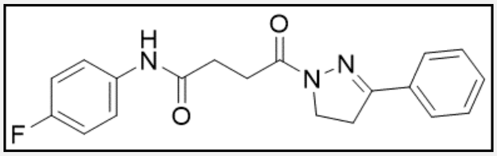	-9.369
ZW-1899	Z146631714	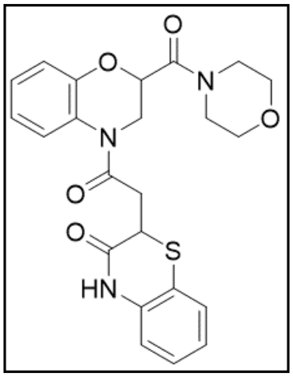	-9.289
ZW-1900	Z729772000	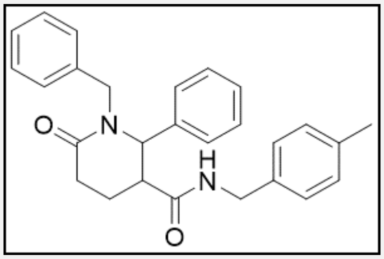	-9.299

ZW-1901	Z811777106	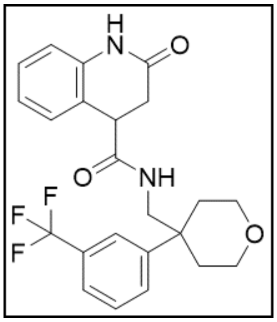	-8.259
ZW-1902	Z1151438917	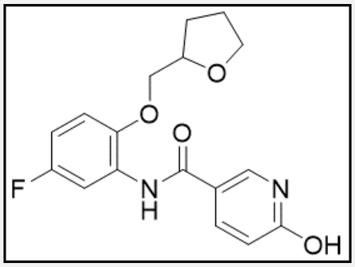	-8.84
ZW-1903	Z228330960	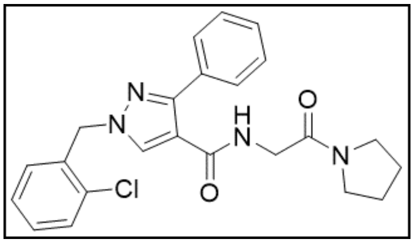	-8.321
ZW-1904	Z107735246	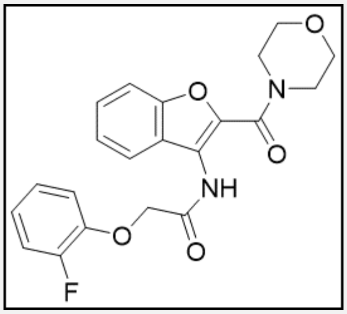	-9.621
ZW-1905	Z89430021	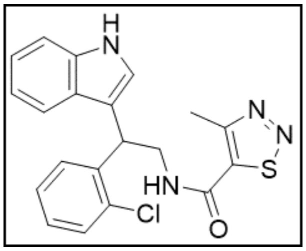	-8.039

ZW-1906	Z223817952	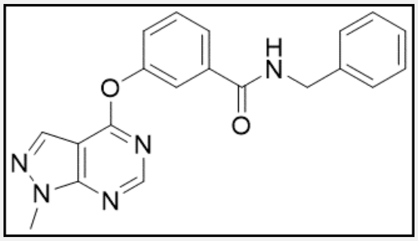	-8.427
ZW-1907	Z30901511	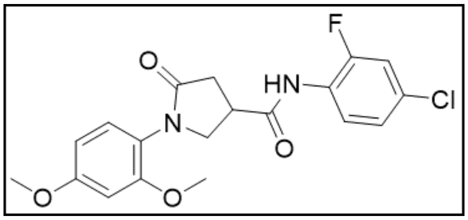	-9.081
ZW-1908	Z225008838	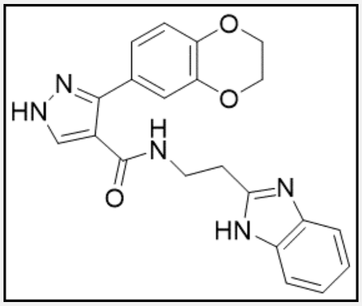	-8.453
ZW-1909	Z300934266	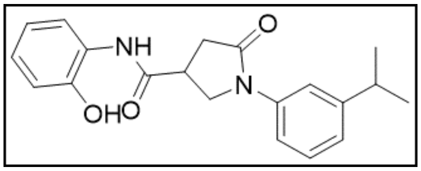	-9.77
ZW-1910	Z30803646	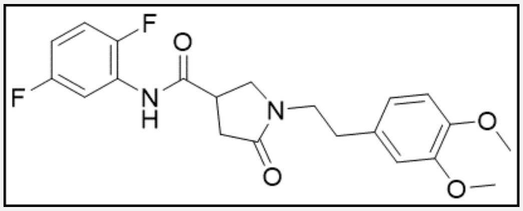	-9.355
ZW-1911	Z317704906	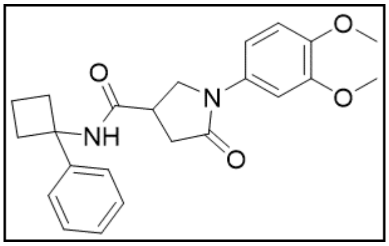	-8.851

ZW-1912	Z105621812	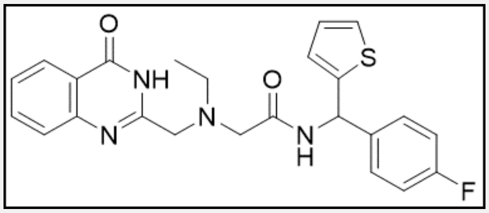	-9.899
ZW-1913	Z65505905	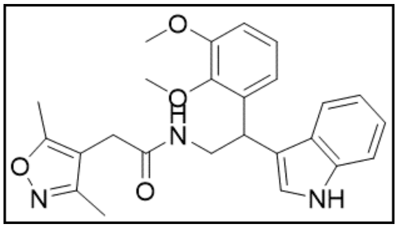	-8.07
ZW-1914	Z241198690	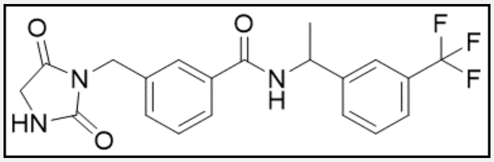	-9.309
ZW-1915	Z91168313	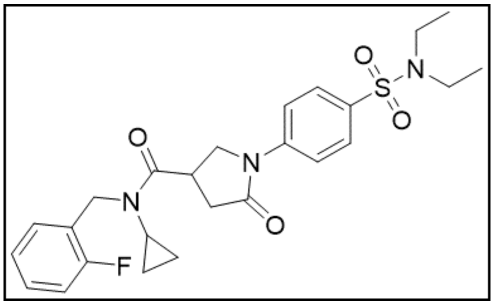	-8.221
ZW-1916	Z295900566	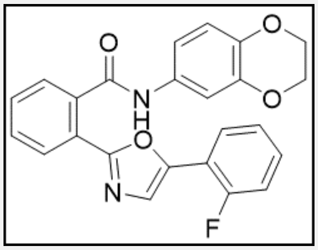	-9.706
ZW-1917	Z337773320	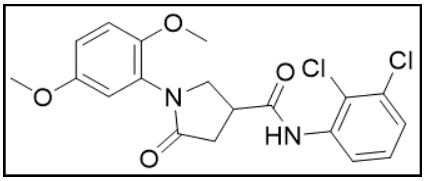	-9.359

ZW-1918	Z109716338	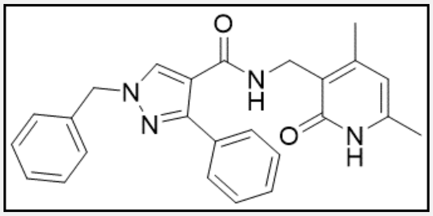	-8.471
ZW-1919	Z1128156429	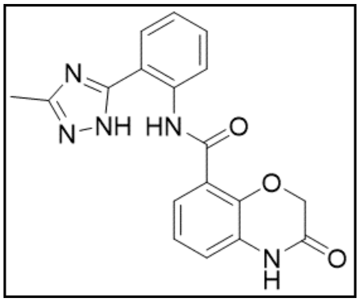	-8.587
ZW-1920	Z1623886528	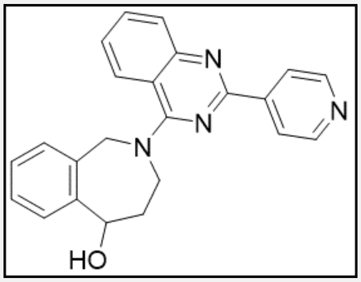	-8.114
ZW-1921	Z1602504897	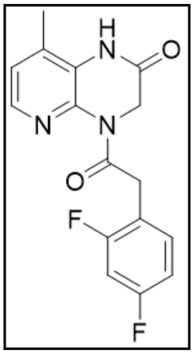	-9.045
ZW-1922	Z728679124	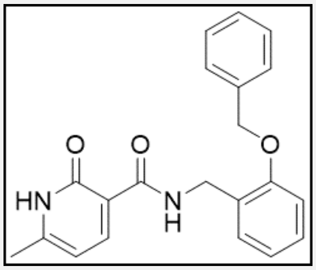	-10.088

ZW-1923	Z1593368890	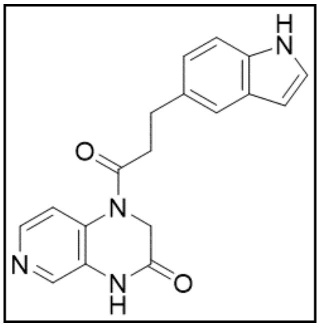	-10.471
ZW-1924	Z367579514	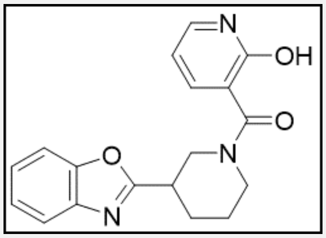	-9.213
ZW-1925	Z73327098	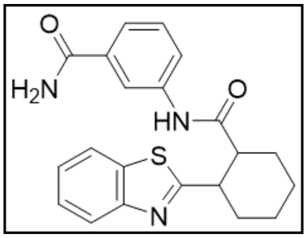	-8.646
ZW-1926	Z90748473	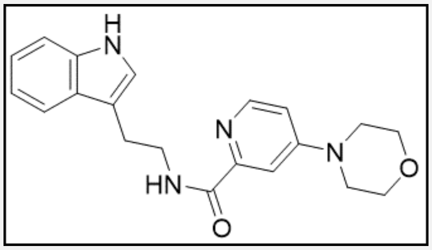	-8.158
ZW-1927	Z1656835879	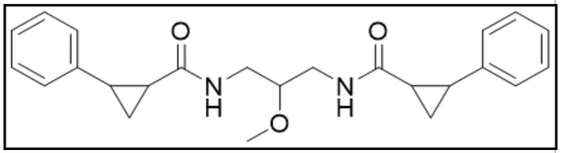	-8.453
ZW-1928	Z30842437	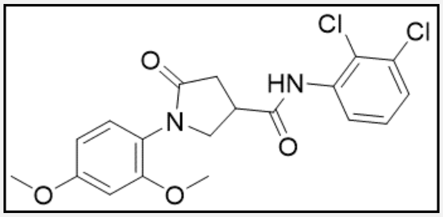	-9.11

ZW-1929	Z57701674	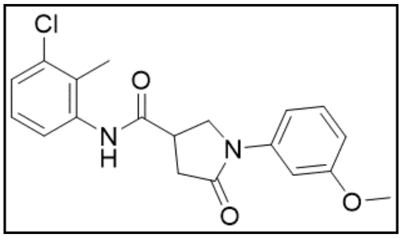	-9.447
ZW-1930	Z57701673	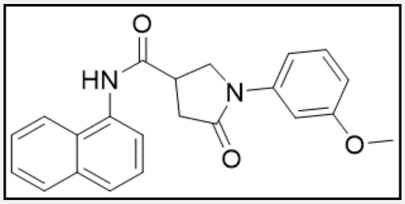	-9.922
ZW-1931	Z1677960943	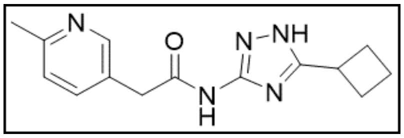	-8.79
ZW-1932	Z1187972693	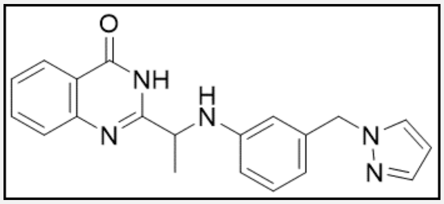	-8.296
ZW-1933	Z1252084076	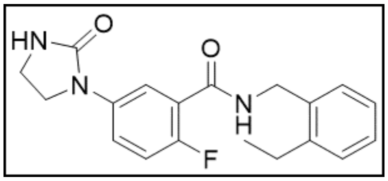	-9.268
ZW-1934	Z1255801905	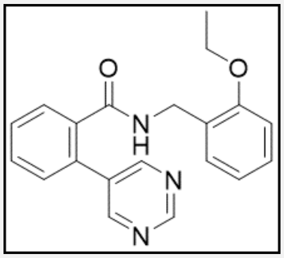	-8.751
ZW-1935	Z1281319177	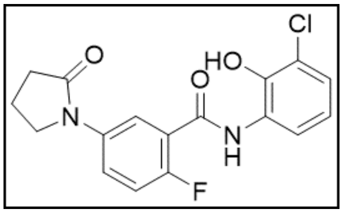	-9.448

ZW-1936	Z300109858	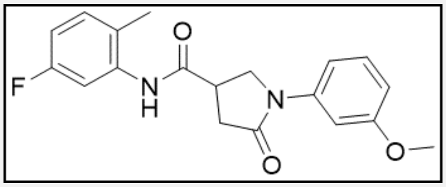	-9.814
ZW-1937	Z235833630	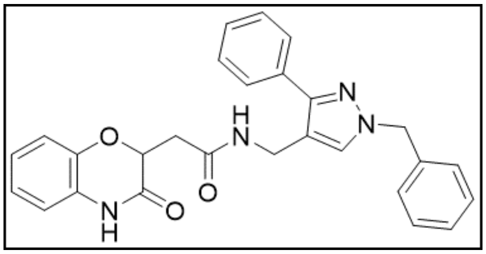	-10.368
ZW-1938	Z336817140	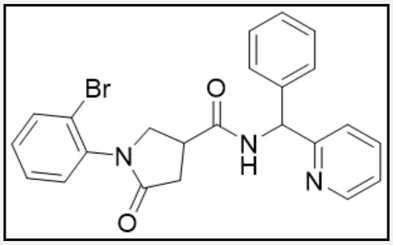	-9.377
ZW-1939	Z30803645	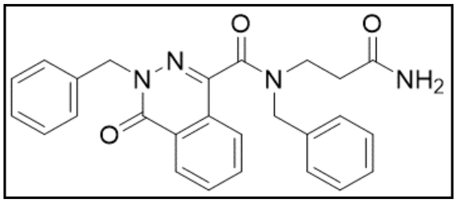	-10.092
ZW-1940	Z1213665994	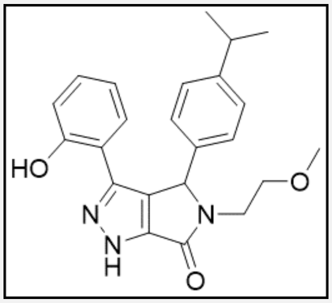	-8.137
ZW-1941	Z729234000	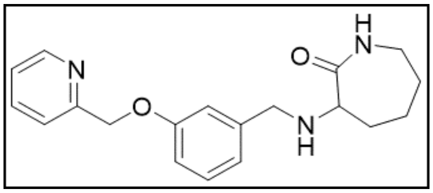	-8.998

ZW-1942	Z85937768	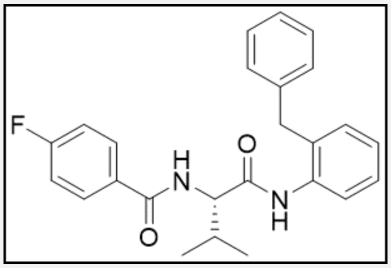	-9.72
ZW-1943	Z1462439959	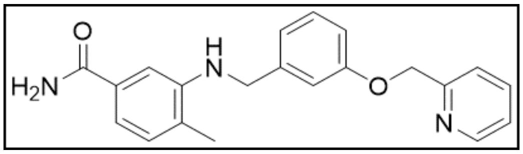	-9.702
ZW-1944	Z200350408	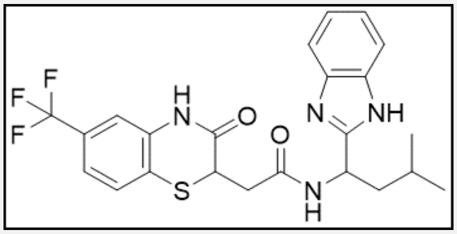	-8.078
ZW-1945	Z50928823	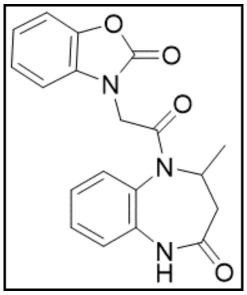	-9.411
ZW-1946	Z241177004	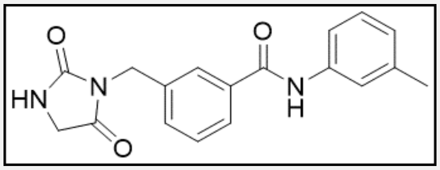	-8.685
ZW-1947	Z1126831590	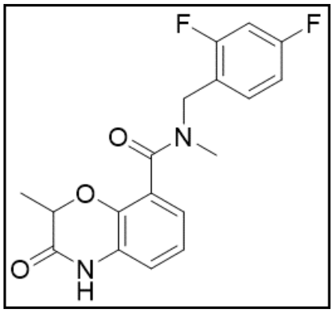	-8.071

ZW-1948	Z1408114867	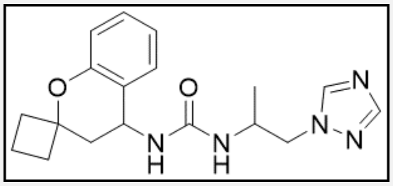	-8.617
ZW-1949	Z57908252	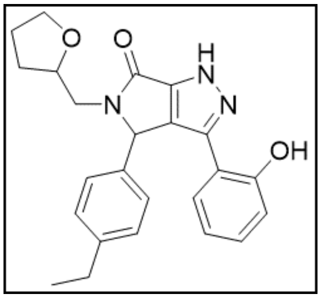	-8.028
ZW-1950	Z1835903985	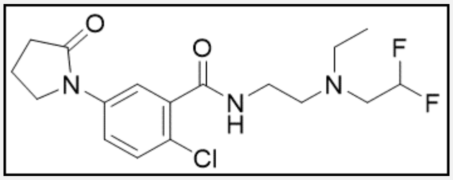	-9.211
ZW-1951	Z1317957310	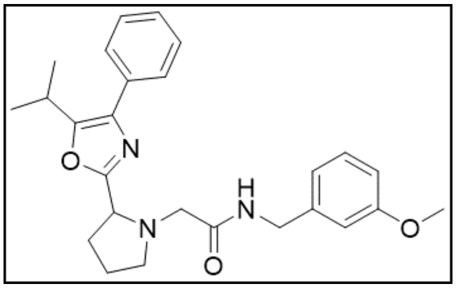	-9.625
ZW-1952	Z319818790	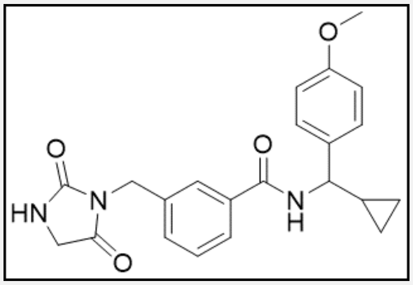	-9.109
ZW-1953	Z166728380	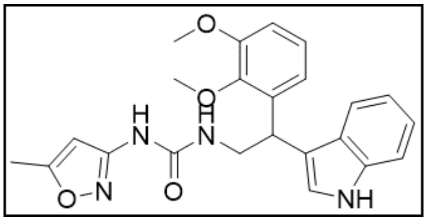	-9.47

ZW-1954	Z905720180	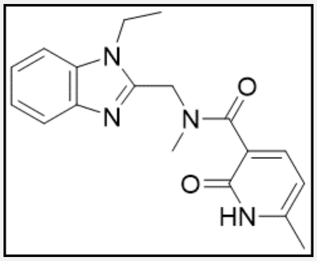	-9.702
ZW-1955	Z651355106	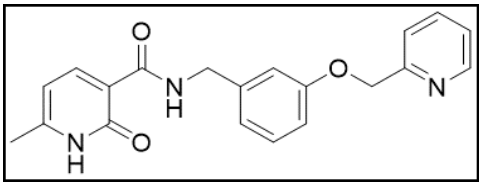	-10.158
ZW-1956	Z99371602	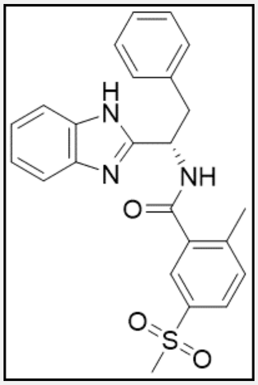	-8.809
ZW-1957	Z30720840	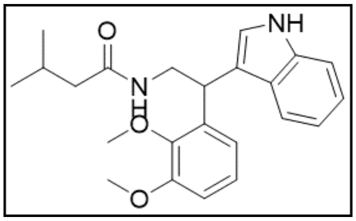	-8.414
ZW-1958	Z234988710	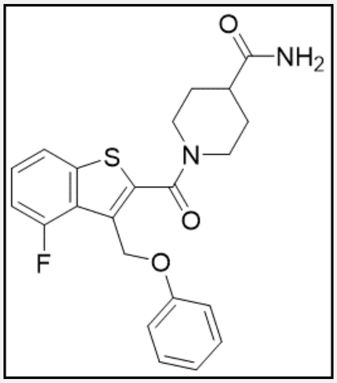	-9.363

ZW-1959	Z408804338	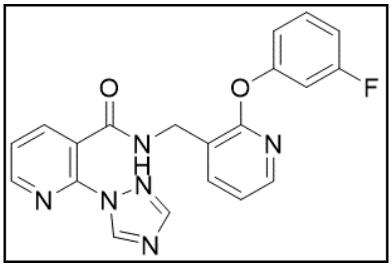	-8.219
ZW-1960	Z296063700	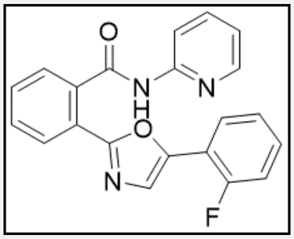	-9.467
ZW-1961	Z408065288	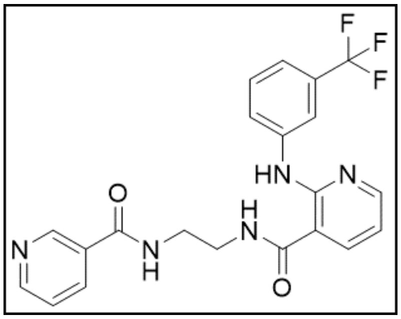	-9.552
ZW-1962	Z412447334	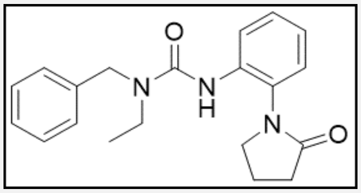	-9.615
ZW-1963	Z229362132	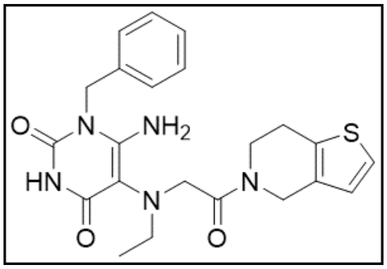	-8.281

ZW-1964	Z102206636	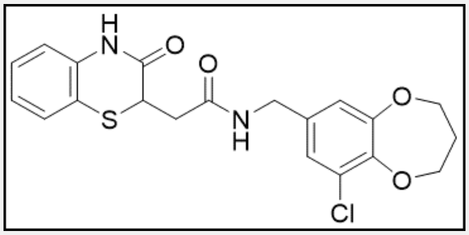	-9.209
ZW-1965	Z167528448	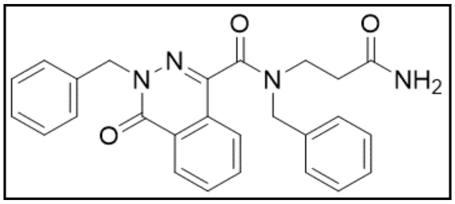	-9.564
ZW-1966	Z236589324	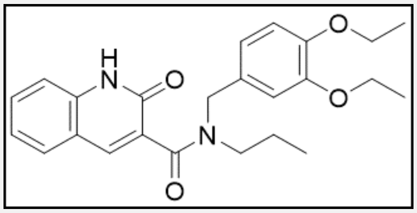	-9.605
ZW-1967	Z97475999	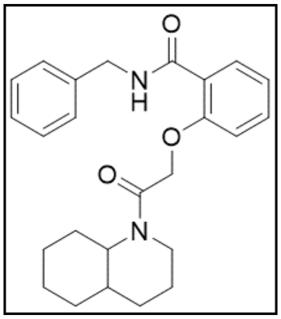	-8.012
ZW-1968	Z29122861	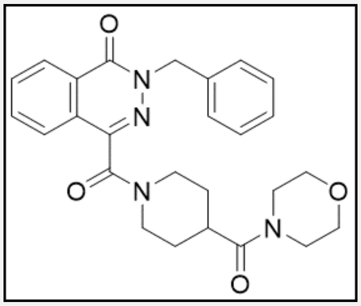	-9.681

ZW-1969	Z27624125	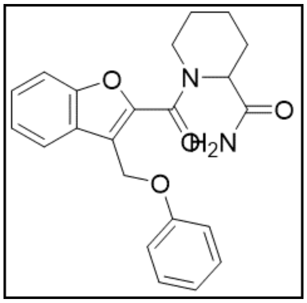	-8.82
ZW-1970	Z31360254	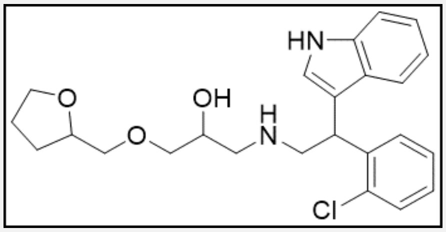	-8.769
ZW-1971	Z1713287452	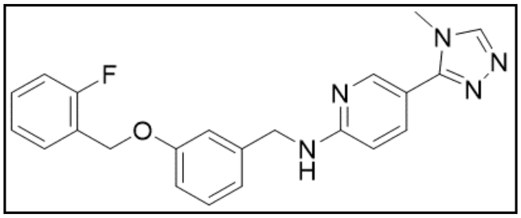	-8.603

Figure S1: Selected TSA curves

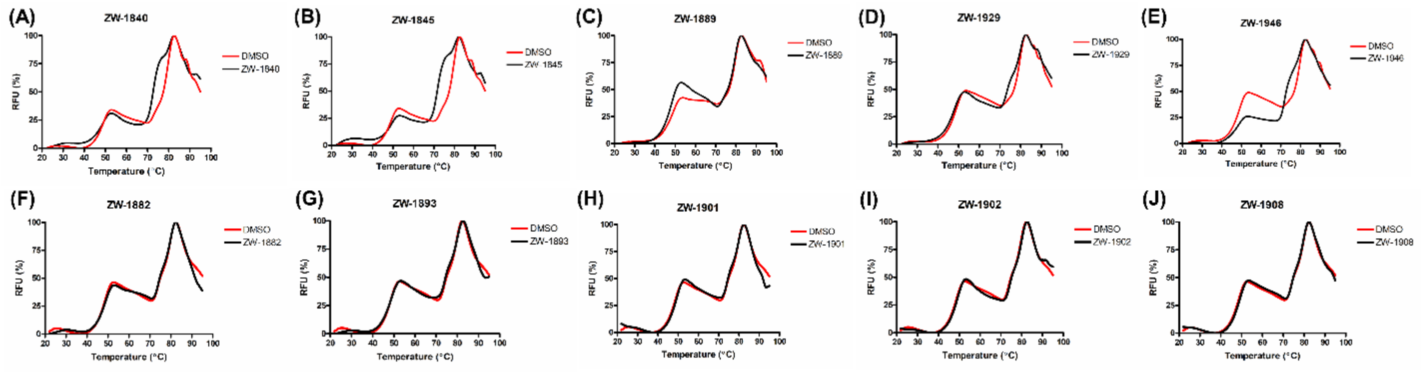

Supplementary Figure 1. (**A-E**) TSA profiles of additional selected hit compounds that bind to HBV Cp and affect capsid formation and stability. (**F-J**) Example TSA profiles of selected compounds screened that did not bind to HBV Cp. HBV Cp melting curves in the presence of 1% DMSO are shown in red; HBV Cp melting curves in the presence of 20 μM compound and 1% DMSO are shown in black.

References

1. Gama, F. H. S.; de Souza, R. O. M. A.; Garden, S. J., An efficient green protocol for the preparation of acetoacetamides and application of the methodology to a one-pot synthesis of Biginelli dihydropyrimidines. Expansion of dihydropyrimidine topological chemical space. *RSC Advances* **2015,** 5, (87), 70915-70928.
2. Abdelrazek, F. M.; Sharaf, M. F.; Metz, P.; Jaeger, A., The reaction of 2-dimethylaminomethylene-3-oxo-N-phenylbutyramide with active methylene nitriles. *J. Heterocycl. Chem.* **2010,** 47, (3), 528-533.
3. Savant, M. M.; Pansuriya, A. M.; Bhuva, C. V.; Kapuriya, N.; Patel, A. S.; Audichya, V. B.; Pipaliya, P. V.; Naliapara, Y. T., Water Mediated Construction of Trisubstituted Pyrazoles/Isoxazoles Library Using Ketene Dithioacetals. *J. Comb. Chem.* **2010,** 12, (1), 176-180.
4. Savant, M. M.; Ladva, K. D.; Pandit, A. B., Facile synthesis of highly functionalized novel pyrazolopyridones using oxoketene dithioacetal and their anti-HIV activity. *Synth. Commun.* **2018,** 48, (13), 1640-1648.
5. Abdelrazek, F. M.; Sobhy, N. A.; Metz, P.; Bazbouz, A. A., Synthetic studies with 3-Oxo-N-[4-(3-oxo- 3-phenylpropionylamino)-phenyl]-3-phenylpropionamide. *J. Heterocycl. Chem.* **2012,** 49, (2), 381-387.
